# Physicomechanical and biological properties of additively manufactured photosensitive resins for provisional crowns using affordable LCD printer

**DOI:** 10.1590/1678-7765-2026-0157

**Published:** 2026-09-21

**Authors:** Marina de Brito Teixeira, Marcelo Pereira Brod, Douver Michelon, Adriana Fernandes da Silva, Evandro Piva, Rafael Guerra Lund, Wellington Luiz de Oliveira da Rosa

**Affiliations:** 1 Universidade Federal de Pelotas Faculdade de Odontologia Departamento de Odontologia Restauradora Pelotas Rio Grande do Sul Brasil Universidade Federal de Pelotas, Faculdade de Odontologia, Departamento de Odontologia Restauradora, Pelotas, Rio Grande do Sul, Brasil.; 2 Universidade Federal de Pelotas Faculdade de Odontologia Departamento de Odontologia Social e Preventiva Pelotas Rio Grande do Sul Brasil Universidade Federal de Pelotas, Faculdade de Odontologia, Departamento de Odontologia Social e Preventiva, Pelotas, Rio Grande do Sul, Brasil.

**Keywords:** Provisional restoration, Additive manufacturing, Photosensitive resins

## Abstract

**Introduction:**

Digital technologies and additive manufacturing are transforming restorative dentistry by enabling the production of highly customized provisional crowns using cost-effective methods.

**Objective:**

This study evaluated the physicomechanical and biological properties of photosensitive resins designed for Digital Light Processing (DLP) printers when used to fabricate provisional restorations with an affordable Liquid Crystal Display (LCD) printer.

**Methodology:**

Three resins - NextDent C&B (ND), Cosmos Temp (CT), and Prizma BioProv (PB) - were tested and compared against bis-acryl (BR) and self-cure PMMA (SA) controls. Evaluated properties included flexural strength, elastic modulus (immediate, 30, and 90 days), Knoop microhardness, radiopacity, surface roughness, water sorption, solubility, and dimensional accuracy. Biological assessment employed L929 fibroblast cell viability at 1, 7, and 14 days. Data were analyzed using One-way or Two-way ANOVA and Tukey’s test (p<0.05).

**Results:**

CT exhibited the highest flexural strength, elastic modulus, and Knoop microhardness, alongside the lowest surface roughness, water sorption, and solubility compared to all other materials. ND showed lower surface roughness than controls but higher water sorption. PB presented mechanical properties comparable to those of the controls, but with higher roughness and solubility. All 3D-printed resins exhibited low radiopacity, and, with the exception of BR, cell viability decreased significantly for all materials up to day 14.

**Conclusion:**

Selected photosensitive resins processed via an affordable LCD printer showed physicomechanical performance comparable to traditional bis-acryl and PMMA resins in fabricating provisional crowns. However, the low radiopacity observed for all 3D-printed materials and the time-dependent decrease in cell viability represent limitations that warrant further investigation.

## INTRODUCTION

Digital technologies have gained increasing attention in dentistry, with three-dimensional (3D) printing - also known as additive manufacturing (AM) - emerging as a transformative technique.^1^ Traditionally, provisional crowns are fabricated using bis-acryl resin and self-cure polymethyl methacrylate (PMMA);^2^ AM now facilitates said production using photosensitive resins by means of a precise layer-by-layer process.^3^ This innovative approach has been steadily replacing traditional manufacturing protocols due to its advantages, including the capability to produce highly customized components that adapt precisely to prosthetic preparations while enhancing workflow efficiency.^4^ Moreover, it offers precise control over details like color, texture, and material layer thickness.^4^ Clinical success of these photosensitive resins depends on various physicomechanical and biological properties such as flexural strength, elastic modulus, microhardness, surface roughness, cytotoxicity, water sorption, and solubility.^6^

Expiration of patents protecting 3D printer manufacturers has significantly improved access to this technology, making it more affordable and accessible to a broader range of clinicians.^7^ The most widely used 3D printers rely on stereolithography (SLA) and digital light processing (DLP) technologies, whose systems operate via a photopolymerization reaction in which photosensitive resins are polymerized by light exposure.^8^ More cost-effective options, such as ones based on Liquid Crystal Display (LCD) 3D printers, are also available. Desktop LCD systems have emerged as a lower-cost alternative to professional DLP dental printers, potentially facilitating the adoption of 3D printing technologies in smaller clinics and laboratories, particularly in low- and middle-income contexts. These SLA-LCD printers incorporate a highly translucent mask that enhances light penetration while reducing costs, further increasing accessibility.^9^ The primary difference between these systems lies in their light source: DLP printers utilize a light projector to project the image, whereas LCD printers employ a liquid crystal screen to create a mask that blocks light emitted from the LED back panel.^10^Besides, DLP 3D printers typically offer significantly higher light intensity compared to LCD printers, and many photosensitive resins have been specifically designed for use with DLP technology.^9^

LCD 3D printers from manufacturers such as Anycubic (China) and Phrozen (Taiwan) have gained widespread popularity worldwide due to their affordability; however, the availability of resins specifically developed for LCD 3D printers remains limited. Consequently, mono-LCD users often rely on DLP-compatible resins. A previous study reported that photosensitive resins designed for DLP 3D printers can also be used in the LCD printer Sonic (Phrozen Sonic, Taiwan) with either a high-powered post-polymerization unit or a low-powered unit applied for an extended duration.^9^ However, evidence regarding whether DLP-designed resins can maintain satisfactory physical, mechanical, and biological properties when processed using LCD-based 3D printers for the fabrication of provisional crowns remains limited. Moreover, it remains unclear whether the performance of these materials is comparable to that of conventional provisional materials, such as bis-acrylic resins and self-curing PMMA.^2^ Thus, this *in vitro* study evaluate the physicomechanical and biological properties of photosensitive resins intended for DLP printers when used to fabricate provisional crowns with an affordable LCD printer (Anycubic, China). Additionally, the study compared the properties of 3D-printed resins with those of bis-acryl resin and self-cure PMMA, positing that 3D-printed resins manufactured with a low-cost LCD printer would exhibit physicomechanical and biological properties comparable to those of bis-acryl and self-cure PMMA resins.

## METHODOLOGY

### Materials used and guidelines

**Figure 1** and **Figure 2** illustrate the experimental study design including the tested resins, printing and post-curing processes, and details of the tested properties. **Figure 3** presents the material types and chemical compositions of the tested resins according to the manufacturers and the polymerization methods. All materials were handled according to the standards recommended by their manufacturers. For the additive manufacturing resins, specimens were designed on specific free and open-source software (TinkerCad, Autodesk, United States, www.tinkercad.com). Bis-acryl resin and self-cure PMMA specimens were created using a stainless steel or a silicone mold (Reflex, Yller Biomateriais, Brazil). A uniform cleaning protocol using 5 minutes of isopropyl alcohol was adopted for all 3D-printed resins to standardize post-processing and allow fair comparisons between materials, as well as to minimize potential adverse effects associated with prolonged washing times previously reported in the literature.^3-5^

**Figure 1:**
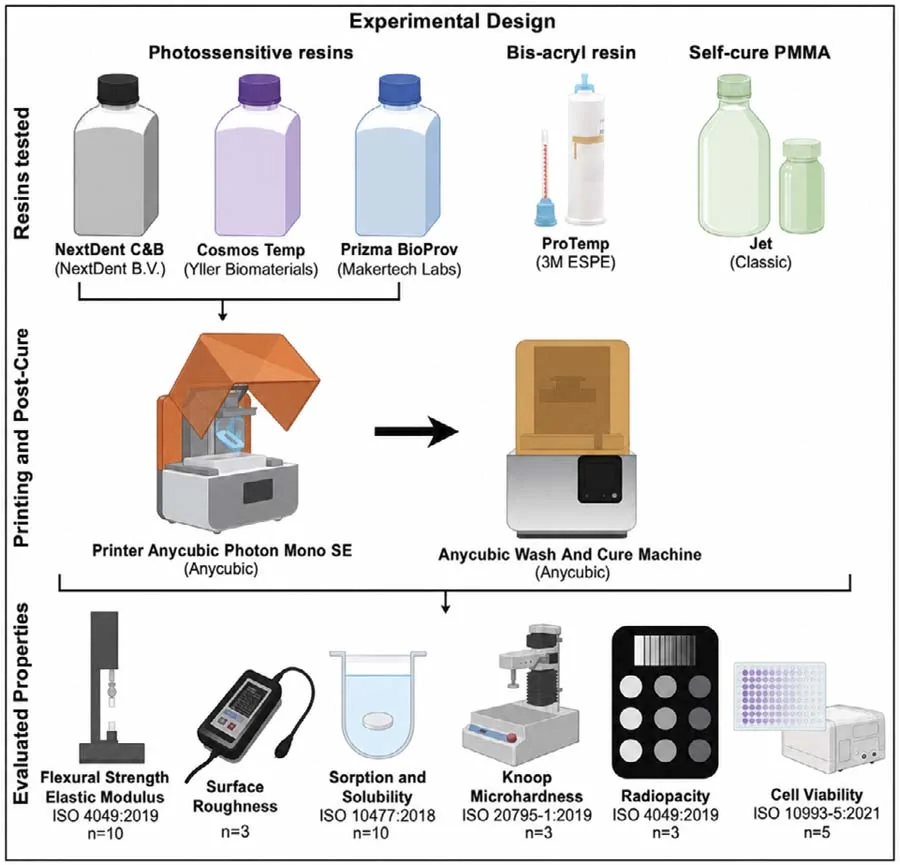
– Experimental design of the study with tested resins, including printing and post-curing processes for photosensitive resins. The figure also details the evaluated properties, corresponding standards, and the number of specimens tested (n). Created with the assistance of BioRender (www.biorender.com).

**Figure 2:**
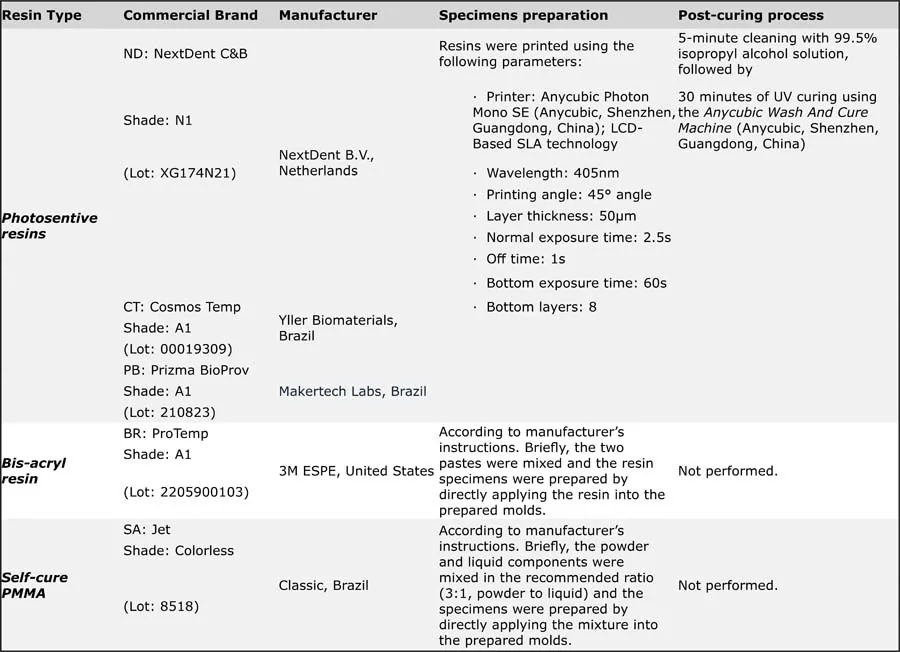
Main characteristics of resins used, including types, brands, manufacturers, specimen preparation, and post-curing processes.

**Figure 3:**
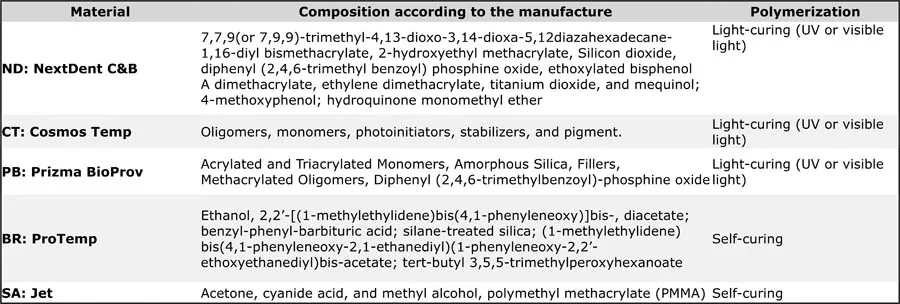
– Materials tested, chemical compositions and polymerization reported by the manufacturers.

Post-curing was performed on a Anycubic Wash and Cure Machine (Anycubic, Shenzhen, China) equipped with 405 nm UV LEDs and a rotating platform. After cleaning, the specimens underwent 30 minutes of UV post-curing at 405 nm with continuous platform rotation. Nominal radiant power of the device is approximately 40 W, as specified by the manufacturer.

Physical properties of the resins used to fabricate provisional crowns were evaluated by the flexural strength, elastic modulus, Knoop microhardness, radiopacity, surface roughness, water sorption and solubility tests. Additionally, cell viability testing was performed to evaluate the biological properties of these materials.

### Evaluation of flexural strength and elastic modulus

Flexural strength and elastic modulus analyses followed the ISO 4049:2019**.**^11^ Although ISO 4049 specifies bar-shaped specimens measuring 25×2×2 mm for three-point bending testing of polymer-based restorative materials, the present study used specimens measuring 64×10×3 mm. These dimensions correspond to those described in ISO 20795-1 for denture base polymers and represent an adaptation widely used in the literature for assessing flexural strength and elastic modulus of provisional crown materials, particularly in studies involving long-term water storage since larger specimens are less prone to handling artifacts and allow more reliable elastic modulus calculations. Each group included ten specimens. One group was tested immediately after preparation and stored in water at 37 ± 1°C for 24 ± 2 h before flexural testing. All remaining specimens were divided into groups (n=10) for long-term assessment and stored in water at 37 ± 1°C for 1 and 3 months before flexural testing. Flexural strength of the specimens was determined using an EMIC MBio2 universal testing machine (EMIC, Biopdi, Brazil). Each specimen was positioned in a steel three-point bending device with a span length of 20 mm and tested at a crosshead speed of 1±0.3 mm/min using a 1 kN load cell.

### Knoop microhardness

Each group included three specimens (10mm in diameter and 1 mm in thickness). Polishing was performed using the Twist-Gloss Diamond Spiral Polisher system (American Burrs, Brazil), following the manufacturer’s recommended speed range (approximately 7,000–10,000 rpm under low pressure, intermittent contact, and without water cooling), for up to 1 minute per specimen face. One operator performed all polishing procedures for protocol standardization. Knoop microhardness was determined for each specimen using a microhardness tester (Future-Tech Corp FM-700, Tokyo, Japan). A load of 50g/force was applied using an indenter with an interval of 10 seconds, in 3 equidistant locations of the specimen, and the average microhardness was determined for each specimen. Analysis was performed according to ISO 20795-1:2013.^12^

### Radiopacity assessment

For each material, three samples (6mm in diameter and 1 mm in thickness) were tested in the radiopacity analysis (ISO 4049:2019).^11^Enamel and dentin samples were used as controls. An occlusal radiographic film (Insight, Kodak Company, NY, USA) was used and the samples were radiographed with an X-ray device (X-Ray AXR – Saevo, Brazil) operating at 70 kV and 7 mA with an exposure time of 0.32 s and a focus-film distance of 30 cm. After processing, the optical density or gray tones of the images were measured and obtained using the ImageJ 1.4 software (National Institute of Mental Health, Maryland, USA). Five points on each specimen were randomly selected to obtain the mean radiopacity value (R) in pixels, which were later transformed into mm Al according to an aluminum scale (from 1.0 mm to 10.0 mm).

### Surface roughness

Surface roughness (Ra) was measured using a digital roughness meter (SJ-201; Mitutuyo, Tokyo, Japan) with a 0.01 μm resolution used at room temperature. Polishing was performed using the Twist-Gloss Diamond Spiral Polisher system (American Burrs, Brazil), following the manufacturer’s recommended speed range (approximately 7,000–10,000 rpm under low pressure, intermittent contact, and without water cooling), for up to 1 minute per specimen face. One operator performed all polishing procedures for protocol standardization. All specimens (n=3, 10 mm in diameter and 1 mm in thickness) underwent the first measurement before polishing, and the second measurement after polishing. Each specimen underwent three readings, and the roughness value (Ra, μm) of each sample was determined from the arithmetic mean of the three measurements.

### Water sorption and solubility evaluation

Each group included ten specimens (5 mm in diameter and 1 mm in thickness) (ISO 10477:2018).^13^ All specimens were weighed after 24 h of polymerization until obtaining a constant initial mass (m1). The samples were then stored in 20 ml of water at 37±1ºC for 7 days. Afterwards, all specimens were washed separately and dried with absorbent paper until free of visible moisture. After the specimens were shaken in air for 15 s and weighed again after 1 min (m2), they were oven-reconditioned until the mass loss of each was less than 0.1 mg for each 24 h period, and the final mass was recorded (m3).  Water solubility(WSL =[(m1−m3)/m3]×100) and  sorption (WSR=[(m2−m3)/m3]×100) were calculated as percentages of the original weight.

### Cell viability assessment

Cell viability analysis was conducted according to ISO 10993-5:2009^14^ using L929 mouse fibroblasts. Cells were cultured in Dulbecco’s Modified Eagle Medium (DMEM) supplemented with 10% fetal bovine serum (FBS), 1% L-glutamine, 100 U/mL penicillin, and 100 µg/mL streptomycin. Fibroblasts were maintained in DMEM and incubated at 37°C in a humidified atmosphere with 5% CO_2_ until sub-confluence was achieved. Cells were seeded at a density of 2x10⁴ cells/well in 96-well plates and incubated under 100% humidity and 5% CO_2_ at 37°C. Prior to the cell viability assay, all specimens were disinfected by immersion in 70% ethanol for 30 s and then exposed to ultraviolet light for 20 min on each side under aseptic conditions. After 1, 7, and 14 days, eluates from 24-well plates containing one specimen per well (6 mm diameter, 1 mm thickness) with 1 mL of medium were used. This eluate was applied to the cells in the 96-well plates for 24 hours. Following incubation, cell viability was assessed using MTT assay (Sigma, USA). After 4 hours of incubation at 37°C in the dark, the blue formazan precipitate formed in the mitochondria was solubilized with 200 µL/well of DMSO. Absorbance was measured at 540 nm using a spectrophotometer (Thermo Fisher Scientific, USA). The untreated group (cell control) was considered equal to 100%. A negative control (cells exposed to culture medium only) was used as a non-cytotoxic reference, and dimethyl sulfoxide (DMSO) at 10% was used as a positive control for cytotoxicity, according to ISO 10993-5:2009.

### Accuracy evaluation

Dimensional accuracy of the 3D printed samples was determined by comparing the measured dimensions with the designed dimensions (10×2 mm). Six disc-shaped specimens (n=6) of each material were used. Diameter and thickness were measured using a digital caliper (0.01 mm accuracy) at three equidistant points per specimen, the mean value of which was used for analysis. Accuracy was calculated as A=((MV−RV)/RV)×100 , in which A represents the accuracy, RV is the reference value, and MV denotes the measured value. This approach provides an indirect estimate of dimensional fidelity in linear dimensions. More comprehensive techniques such as digital scanning superimposition or micro-CT analysis would allow a three-dimensional evaluation of internal and marginal fit, and this limitation is addressed in the Discussion section.

### Statistical analysis

Data were analyzed on SigmaPlot 12.0 software (Systat Software, Inc., United States). Normality and homogeneity of sample variance were assessed by the Shapiro-Wilk and Levene tests. Flexural strength, elastic modulus and cell viability were evaluated using Two-Way ANOVA followed by Tukey’s post-hoc test. Hardness and radiopacity were analyzed using One-Way ANOVA followed by Tukey’s post-hoc test. Surface roughness was evaluated considering before and after polishing data with Two-Way Repeated Measures ANOVA test followed by Tukey’s post-hoc test. Water sorption and solubility were assessed by the Kruskal-Wallis test followed by Dunn’s post-hoc test. Sample size for each test was defined based on previous studies that evaluated similar physicomechanical and biological properties of 3D-printed and conventional provisional resins, and on the requirements specified by the corresponding ISO standards. A significance level of 5% was adopted.

## RESULTS

### Flexural strength and Elastic Modulus

3D-printed resins outperformed the self-cure acrylic resin (p<0.05) in the three-point bending test (**Figure 4a**). At all points, CT presented the highest flexural strength among all tested resins, with the maximum value recorded as 112.7 (±11.4) MPa after 30 days (p<0.05). Overall, the 3D-printed and BR resins showed an increase in flexural strength after 30 days (p<0.05), with values returning to levels statistically similar to the immediate measurements after 90 days (p<0.05). ND and PB resins were comparable to the bis-acrylic resin control at all time points, consistently exhibiting flexural strength values exceeding the 50 MPa minimum recommended by ISO 4049:2019.^11^

**Figure 4:**
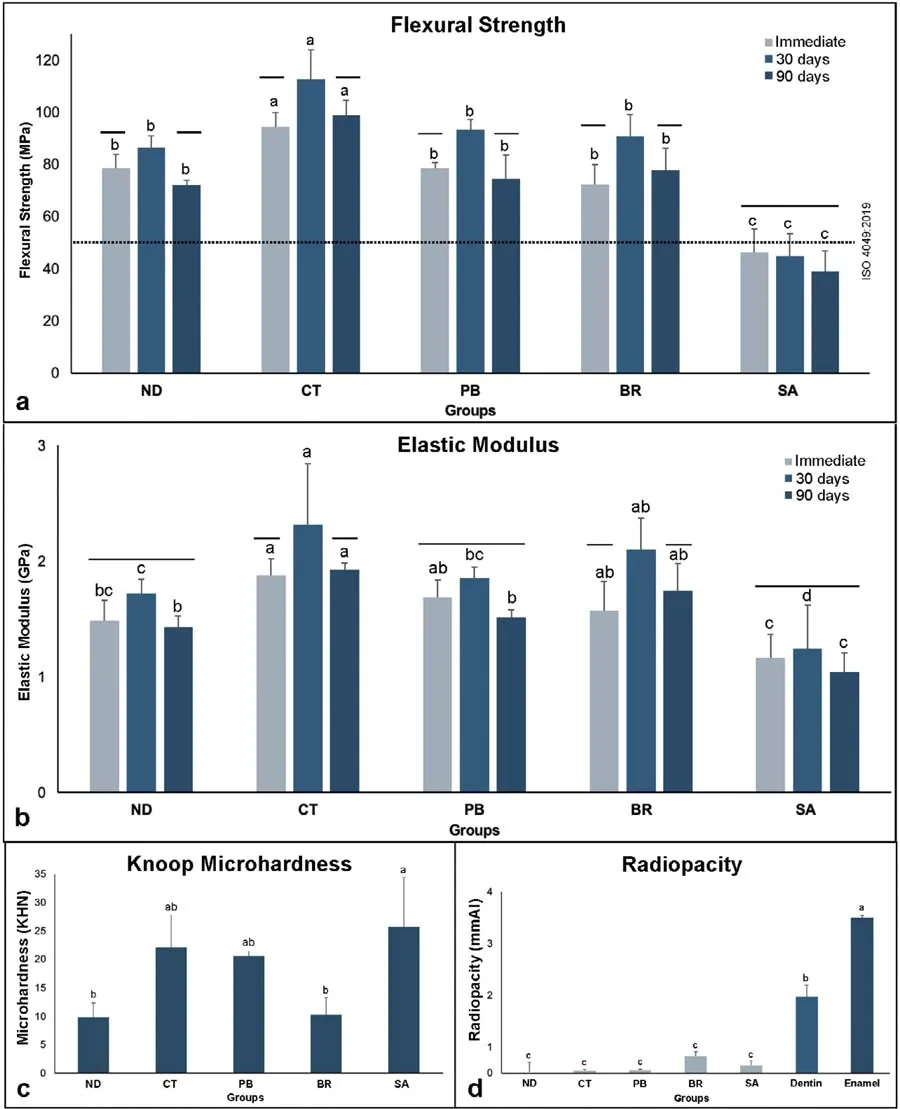
– Test results regarding: a) Flexural Strength at Immediate, 30-Day, and 90-Day intervals. Lines at the same height indicate no statistically significant differences for the same resin at different time intervals (p>0.05). Different lowercase letters indicate statistically significant differences among different resins at the same time point (p<0.05). The dotted line represents the ISO 4049:2019 recommended parameter of at least 50 MPa flexural strength for temporary materials. b) Elastic Modulus at Immediate, 30-Day, and 90-Day intervals. Lines at the same height indicate no statistically significant differences for the same resin at different time intervals (p>0.05). Different lowercase letters indicate statistically significant differences among different resins at the same point (p<0.05). c) Knoop Hardness of all groups tested. Lowercase letters indicate statistically significant differences among resins (p<0.05). d) Radiopacity of resin groups compared to dentin and enamel substrates. Lowercase letters indicate statistically significant differences among groups (p<0.05).

Elastic modulus of the ND, PB, and SA resins remained unaffected by the evaluation period (**Figure 4b**), whereas CT and PB exhibited a higher elastic modulus after 30 days. CT showed the highest elastic modulus among all 3D-printed resins and conventional self-cure PMMA at both 30 and 90 days (p<0.05). After 90 days, ND and PB presented elastic modulus values statistically comparable to the BR control group, both of which were higher than the self-cure PMMA resin (p<0.05).

### Knoop microhardness

CT and PB were the 3D resins with the highest values in the Knoop microhardness test (**Figure 4c**), 22.14 (±5.74) and 20.56 (±1.13) KHN, respectively, which were comparable to the self-cured PMMA control group (SA: 25.77 ± 8.77 KHN). ND presented similar values (9.84 ± 2.50 KHN) to the other 3D resins and the bis-acrylic resin control group (BR: 10.33 ± 3.04 KHN), but significantly lower than the self-cured acrylic resin control group (SA) (p<0.05).

### Radiopacity

All tested 3D resins showed radiopacity (**Figure 4d**) lower than 0.06±0.02mm Al (PB) and were statistically similar to the controls with bisacrylic (BR: 0.32±0.09mm Al) and self-cure acrylic (SA: 0.15±0.10mm Al) resins (p>0.05). All groups presented radiopacity significantly lower than the enamel (3.01±0.04) and dentin (1.48±0.22) controls (p<0.05).

### Surface roughness assessment

Before polishing, CT exhibited the highest surface roughness (4.23 ± 0.14 Ra) followed by ND (3.78±0.29 Ra) and PB (2.84±0.10 Ra) (**Table 1**). All 3D-printed resins presented significantly higher surface roughness compared to both control groups (p<0.05). After the polishing protocol, ND and CT showed a significant reduction in surface roughness (p<0.05), achieving the lowest values of 0.75±0.05 Ra and 0.77±0.01 Ra, respectively. Both ND and CT were statistically different from the control groups after polishing (p<0.05). Conversely, PB showed no improvement in surface roughness after polishing and maintained the highest roughness value among all groups.

**Table 1:** – Mean and Standard Deviation (±) of Surface Roughness (Ra) before and after polishing, Median and Interquartile Ranges for Surface Roughness, Sorption, and Solubility Test Results, Mean and Standard Deviation (±) of the diameter accuracy evaluation.

Resin	Surface Roughness (Ra) (n=3)		Water Sorption (%) (n=10)	Water Solubility (%) (n=10)	Accuracy (%) (n=6)
	Before	After			
**ND: NextDent C&B**	3.78 (±0.29)^Aa^	0.75 (±0.05)^Ab^	46.60 (44.43-48.01)^A^	1.51 (1.16-2.67)^AB^	-0.95 (±0.59) ^c^
**CT: Cosmos Temp**	4.23 (±0.14)^Ba^	0.77 (±0.01)^Ab^	15.12 (12.62-15.52)^C^	0.74 (0.38-1.27)^B^	0.07 (±0.22) ^c^
**PB: Prizma BioProv**	2.84 (±0.10)^Ca^	2.74 (±0.03)^Ca^	33.41 (31.94-34.01)^AB^	5.14 (3.19-6.11)^A^	2.09 (±0.27) ^b^
**BR: ProTemp**	1.18 (±0.01)^Da^	1.15(±0.02)^Bb^	16.72 (14.32-20.92)^C^	1.25 (1.10-1.27)^B^	3.89 (±1.76) ^ab^
**SA: Jet**	1.38 (±0.18)^Ea^	1.40 (±0.02)^Ba^	24.03 (22.61-26.23)^BC^	1.35 (0.77-1.67)^B^	5.71 (±2.14) ^a^

Surface Roughness (Ra): Different capital letters in the same column indicate statistically significant differences between resins, and different lowercase letter in the same line indicate statistically significant differences before and after polishing (p<0.05). Mean and Standard Deviation are shown for parametric statistics using the Two-Way Repeated Measures ANOVA test followed by Tukey’s post-hoc test.

Water Sorption (%) and Solubility (%): Different letters in the same column indicate statistically significant differences between resins (p<0.05). Median and Interquartile Ranges are shown for non-parametric statistics using the Kruskal-Wallis test followed by Dunn’s post-hoc test.

### Sorption and solubility

Regarding water sorption assessment (**Table 1**), CT achieved the lowest sorption value among the 3D-printed resins at 15.12% (12.6–15.5), similar to the controls—bis-acrylic resin at 16.72% (14.32–20.92) and self-cure acrylic resin at 24.03% (22.61–26.23) (p>0.05). ND exhibited the highest sorption value of 46.60% (44.43–48.01), which was statistically different from the BR and SA controls (p<0.05). PB presented a sorption value of 33.41% (31.94–34.01), similar to ND and the SA control.

Concerning water solubility (**Table 1**), all results for the 3D resins varied when compared to the controls. PB achieved the highest solubility value at 5.14% (3.19–6.11), which was statistically different from both controls (p<0.05). Conversely, ND and CT showed solubility values similar to the BR and SA controls (p>0.05).

### Accuracy Evaluation

Dimensional accuracy evaluation results (**Table 1**) revealed that CT and ND resins achieved the smallest mean deviation from the reference dimension, with values of 0.07 (±0.22)% and −0.95 (±0.59)%, respectively, (p>0.05). Both resins also performed statistically better than the other groups. PB presented an accuracy of 2.09 (±0.27) %, whereas SA and BR resins exhibited mean accuracies of 5.71 (±2.14)% and 3.89 (±1.76)%, respectively. PB and BR were statistically similar to each other, and SA was statistically similar to BR.

### Cell viability

**Figure 5** shows the percentage of cell viability evaluated after 1, 7, and 14 days. After 24 hours, all 3D resins exhibited higher cell viability results which were statistically significantly different from those found at 7 and 14 days (p<0.05), indicating a decrease in cell viability over time. After 14 days, the 3D resins presented lower cell viability statistically different from the cell control (p<0.05). Specifically, ND achieved 59.63% (±31.71) cell viability after 14 days, a value statistically different from both the cell control and the BR control at the same time point (p<0.05). Similarly, CT showed a cell viability of 67.79% (±14.52) after 14 days, which was statistically different from the cell control (p<0.05). ND and CT were the only resins considered cytotoxic after 14 days, as their cell viability was below the 70% threshold recommended by ISO 10993-5:2009.^14^

**Figure 5:**
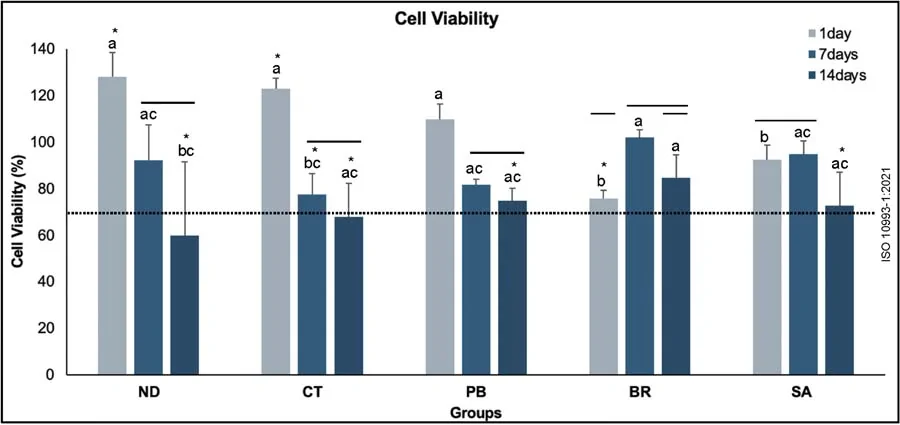
– Cell Viability of L929 Fibroblasts after 1, 7, and 14 Days. Lines at the same height indicate no statistically significant differences for the same resin at different time intervals (p>0.05). Different lowercase letters indicate statistically significant differences among resins at the same time point (p<0.05). The dotted line represents the ISO 10993-5:2021 recommended parameter of at least 70% cell viability for a non-cytotoxic material. An asterisk (*) indicates statistically significant differences between resins and the cell control at the same time point (p<0.05).

## DISCUSSION

The physicochemical and biological properties of 3D-printed resins are critical to their performance in the oral cavity. As new materials for additive manufacturing continue to emerge, it is essential to evaluate their properties and compare them to those of traditional materials used for provisional crowns to predict the clinical performance of photosensitive resins. This study investigated various properties of resin materials used in the additive manufacturing of provisional crowns with a low-cost SLA-LCD printer, comparing them to bis-acrylic and self-cure PMMA resins. Study findings revealed that some of the 3D-printed resins achieved physical and biological properties comparable to those of the tested bis-acrylic and self-cure acrylic resins, supporting the research hypothesis. However, the results varied depending on the specific 3D resin and the property assessed.

Evaluating DLP-designed resins processed in an LCD printer is clinically meaningful, since the limited commercial availability of resins specifically marketed for LCD systems forces many clinicians and laboratories to use DLP-compatible materials in LCD devices. The two technologies, however, differ in important aspects regarding their light source: DLP printers use a digital micromirror device coupled to a projector that typically delivers higher light intensity, whereas LCD printers rely on an LED back panel filtered through a liquid-crystal mask, which generally results in lower irradiance reaching the resin vat. The wavelength emitted by most current LCD systems (around 405 nm) is, however, compatible with the photoinitiators (mainly TPO and BAPO derivatives) used in DLP-oriented resins which supports their cross-use. Here, this potential mismatch in irradiance was compensated by adjusting layer exposure times and by applying a standardized post-curing protocol (Anycubic Wash and Cure Machine, 30 min), which has been previously suggested as a strategy to achieve adequate degree of conversion when DLP resins are processed in LCD devices. This contextualization helps interpret the heterogeneous behavior observed across the tested resins, since materials originally optimized for high-irradiance DLP exposure may not reach identical conversion levels in an LCD workflow, potentially influencing mechanical, sorption, and biological outcomes.

Regarding mechanical properties, the 3D-printed resins ND and PB performed similarly to the bis-acrylic resin (control) during the 90-day evaluation period, exhibiting the highest flexural strength at 30 days. Results at 30 and 90 days followed consistent performance patterns, indicating that the flexural strength and elastic modulus of these materials remain stable over time. Interestingly, the increase in flexural strength observed after 30 days may be associated with different complementary mechanisms. First, continued late-curing/post-curing reactions during water storage at 37 °C may further increase the degree of conversion of unreacted methacrylate groups. Additionally, structural relaxation of the polymeric network and reduction of internal stresses generated during the layer-by-layer photopolymerization process may contribute to improved mechanical behavior over time. Water sorption may also play a dual role - although water can act as a plasticizer, moderate uptake during the initial storage period may help redistribute internal stresses without causing significant polymer matrix degradation. Return to values similar to the immediate condition after 90 days is consistent with the onset of hydrolytic effects on the polymeric matrix previously described in the literature. For the controls, a previous study assessing the flexural strength of conventional PMMA resins reported a similar range of values to those found here, varying from 49 (±5.8) MPa immediately after fabrication to 69 (±6.7) MPa after aging.^1^ Consistent with the present findings, another study^16^ demonstrated that the bis-acrylic resin exhibited significantly higher flexural strength (131.9±5.8 MPa) compared with conventional PMMA systems (66.1±13.1 MPa).

Various factors can influence the mechanical properties of 3D-printed resins, including printing parameters, layer thickness, postprocessing methods, printing orientation, and slicing configuration.^7,17^A previous study printed CT using an LCD printer and found an immediate flexural strength of 52 (±5.8) MPa, with aged samples showing a statistically similar value of 51 (±5.8) MPa.^15^ Here, CT achieved the highest flexural strength among all tested materials at all evaluated time points. Despite a lack of consensus on the optimal printing angle for 3D resins - since different flexural strength, hardness, and surface roughness results have been reported for printing angles of 0°, 30°, 45°, and 90° ^18-20^ - a systematic review suggested that a 45° print angle provides satisfactory marginal and internal adaptation along with adequate flexural resistance.^2 1^ Thus, we used a printing angle of 45° and a post-curing time of 30 minutes, which may explain the higher flexural strength found for CT using the LCD printer than in previous research.^15^ Additionally, studies have indicated that longer post-curing times can enhance the flexural strength, elastic modulus, and hardness properties of photosensitive resins.^22,23^ Other studies have reported flexural strengths exceeding 80 MPa for ND, CT, and PB, using different printing and postpolymerization methods,^19,23,24^ which are comparable to the results we achieved with an affordable LCD printer.

Post-curing represents a critical step for enhancing the microhardness of 3D-printed resins, as highlighted in previous research.^23^ Thus, we standardized the post-curing process for all 3D-printed resins to enable comparisons between materials produced using an affordable LCD printer. Another study reported a Knoop microhardness for ND of 9.91 ± 3.71 KHN,^25^ consistent with the value obtained here (9.84±2.50 KHN). The same study found no significant differences in Knoop microhardness between ND and bis-acryl resin.^25^ While our study found no differences between CT and PB, a previous study reported a Knoop microhardness of 8.5 (±2.0) KHN for CT and 17.6 (±1.1) KHN for PB, using printing parameters similar to ours.^18^ Unfortunately, a more detailed analysis of the KHN results based on the material composition was not unfeasible since the 3D-printed resin manufacturers closely guard the primary formulations of their products. This confidentiality restricts the ability to identify common patterns regarding monomeric content, photoinitiators, and particle composition that could explain some differences found between materials.

Radiopacity of a resin is essential to ensure that the material does not interfere with imaging exams conducted during patient monitoring to detect potential complications like infiltration, recurrent caries, fractures, or interface failures.^26^ Here, all tested resins were radiolucent, exhibiting lower radiopacity than enamel and dentin. This finding has direct clinical relevance, since the low radiopacity observed for all 3D-printed resins may compromise the radiographic identification of restoration margins, the detection of excess material in the gingival sulcus, and the diagnostic interpretation of recurrent caries, secondary defects, or open margins around the provisional crown. According to ISO 4049, restorative polymer-based materials should exhibit radiopacity equivalent to or greater than that of the same thickness of aluminum—which was not achieved by any of the tested 3D-printed resins. This limitation should be clearly considered when selecting these materials for clinical use, particularly in cases requiring longer-term provisional restorations or radiographic follow-up. While radiopacity is a critical property for provisional materials to facilitate radiographic diagnosis, its absence may also allow direct visualization of the underlying dental substrate without interference from the restorative material in specific situations. Additionally, optimizing the balance between radiopacity and material resistance remains challenging, as the inclusion of radiopaque particles may compromise the mechanical properties of the material.^27^ Further research is needed to evaluate the incorporation of radiopacifying agents such as barium, zinc, or bismuth, in 3D-printed resins. The ongoing technological advancements in 3D printing techniques and the development of innovative materials hold promise for enhancing the radiological imaging capabilities of photosensitive resins.^28^

Surface roughness has been reported to influence bacterial adhesion and fungal activity in 3D-printed resins used for dental crowns and denture bases.^6,29,30^ This highlights the importance of achieving a smooth surface for temporary crowns, as rough surfaces are more prone to staining and can serve as breeding grounds for bacteria, potentially leading to gingival tissue inflammation.^6^ Ra parameter is commonly used to evaluate surface quality by measuring the amplitude of deviations from the sample’s central plane.^31^ Before polishing, all 3D-printed resins exhibited higher surface roughness compared with the controls. One factor that could potentially influence this result is the printing angle of 45°; however, previous research indicated that this specific printing angle does not affect the surface roughness of CT and PB resins.^32^ Regarding the controls, the higher surface roughness observed for the self-cure PMMA group before polishing can be attributed to bubble formation during the hand mixing of the powder and liquid components.^33^ Conversely, bis-acryl resin is manipulated using a dispenser and mixing tips, which promotes greater material homogeneity.^33^

Polishing, therefore, plays a critical role in achieving a smoother surface. Of clinical relevance, Bollen, Lambrechts, and Quirynen^47^ (1997) established a threshold Ra value of 0.2 µm for intraoral hard surfaces, below which no further reduction in bacterial accumulation is expected. Here, even after polishing the Ra values of all tested materials remained above this threshold (ND: 0.75 µm; CT: 0.77 µm; PB: 2.74 µm), indicating that bacterial colonization risk was not eliminated by the polishing protocol applied. This underscores the need for further optimization of surface finishing procedures for 3D-printed provisional resins. Additional surface treatments such as glaze application can also be utilized to further reduce surface roughness and enhance wear resistance.^34^

Following polishing, ND and CT resins showed lower roughness compared with the controls, whereas PB resin exhibited higher values under the applied polishing protocol. This protocol was selected based on a pilot experiment with these resins, which demonstrated that a smoother surface could be achieved in 3D-printed resins using the Twist Gloss Kit (American Burrs, Brazil). Further studies are needed to investigate alternative methods for the PB resin to achieve a smoother surface.

While water sorption can be defined as a diffusion-controlled process occurring within the resin matrix, water solubility is influenced by oral cavity fluids, particularly water, which may contribute to resin degradation.^35^ Our results showed that only the photosensitive resin CT exhibited water sorption and solubility levels comparable to the controls, including bis-acryl resin and conventional PMMA. Conversely, ND presented the highest water sorption, whereas PB achieved the highest solubility. A potential factor influencing water solubility may be the washing time.^35^ We standardized this parameter using a 5-minute cleaning protocol, based on previous findings that extending the washing time from 3 minutes to 1 hour may reduce mechanical properties and increase material solubility.^35^ Previous studies have also reported higher water sorption and solubility in 3D-printed denture base resins, which were also more susceptible to aging.^36,37^Similar results were observed in photosensitive resins used for provisional crowns, which exhibited inferior physical properties and reduced color stability.^38-40^ Thus, CT was the only 3D-printed resin comparable to the controls in terms of water sorption and solubility. Further studies are warranted to assess whether this resin can achieve better color stability than the others.

Materials with high biocompatibility are vital for ensuring the safety and success of dental treatments.^41,42^ After 24 hours, none of the photosensitive resins showed signs of cytotoxicity, aligning with findings from studies on other types of 3D-printed resins.^9,29,43,44^ Previous studies have reported that without post-polymerization, photosensitive resins can exhibit cell viability below 70%, classifying them as cytotoxic.^9^ Additionally, post-curing duration has been shown to affect the cytotoxicity of ND resins on fibroblast cells, with a 15-minute post-polymerization process achieving nearly 100% cell viability.^9^ This highlights the critical role of the material’s degree of conversion in determining its cytotoxicity, as unreacted monomers can have significant biological effects.^29^ Here, a 30-minute post-polymerization protocol was employed, as recommended by the NextDent manufacturer. Over time, all 3D-printed resins exhibited a decline in cell viability, likely due to the release of potentially toxic components into the surrounding media. To the best of our knowledge, this is the first study to demonstrate a potential cytotoxic effect of these materials over a 14-day period.

Prolonged exposure to substances released from dental materials may increase the risk of oral lesions and delayed-type hypersensitivity in susceptible individuals.^45^ Of the 3D-printed resin groups, only PB maintained cell viability above 70% after 14 days and was not classified as cytotoxic according to ISO 10993-5:2009^14^ at this time point. The observed variation in cell viability over time suggests that the biocompatibility of these resins is not static and depends on both the intrinsic properties of the materials and exposure duration. Importantly, the biological interpretation of the present findings is limited to the *in vitro* indirect-contact model used, since L929 mouse fibroblasts represent a standardized initial screening system recommended by ISO 10993-5:2009 but do not reproduce the complexity of the oral environment, including epithelial barriers, salivary clearance, and immune-cell interactions. Additionally, factors related to the experimental model itself—such as changes in cell confluence and metabolic activity over the evaluation period—may also have contributed to the time-dependent reduction in MTT signal observed across all groups, and not exclusively the release of cytotoxic components. As the present study did not include direct quantification of residual monomer release or degree of conversion, the biological outcomes should be interpreted as exploratory and complementary to physicomechanical data. Further studies using degree-of-conversion analysis, chromatographic identification of eluted monomers, and more clinically relevant cell models (e.g., human gingival fibroblasts, oral keratinocytes, or organotypic mucosa) are needed to better characterize the long-term biocompatibility of these materials.

Accuracy of provisional restorations involves a complex interplay of several factors, including material composition, internal design, and manufacturing parameters.^39^ Specific resin properties and manufacturing method critically influence the final dimensional fidelity of the printed object.^46^ Achieving high-fidelity 3D-printed dental applications requires detailed consideration of these interconnected factors to mitigate the shrinkage and warpage inherent to the light-curing process.

Overall accuracy of printed resins can vary depending on the material, with some resins showing an overall accuracy significantly superior to that of other materials. For example, Cosmos presented an overall accuracy similar to the milled PMMA control and superior to other impression resins tested.^18^ Such material-dependent variation, such as the presence and nature of filler particles in the resin, also plays an important role in material selection to achieve the desired accuracy in provisional restorations. This highlights the importance of considering not only the resin properties but also how these characteristics influence the fit and accuracy of the printed components, a point emphasized by the study results which reported a significant variation in part fit.

Results showed a variation in dimensional accuracy across the tested materials. When the percentage deviations were translated into absolute linear deviations from the reference dimension of 10 mm, the 3D-printed resins showed mean deviations of approximately 7 μm (CT), 95 μm (ND), and 209 μm (PB), whereas the conventional control groups exhibited larger deviations (approximately 389 μm for BR and 571 μm for SA). Although some 3D-printed values exceed the 120 μm clinical limit classically suggested by McLean and von Fraunhofer^46^ for definitive restorations, the 3D-printed resins still achieved a closer fit to the designed dimensions than the bis-acryl and self-cure PMMA controls. Importantly, however, this evaluation was based on linear caliper measurements of disc-shaped specimens which provides only an indirect estimate of dimensional fidelity. More comprehensive approaches, such as 3D scanning superimposition or micro-CT analysis of crown-shaped specimens cemented over standardized abutments, are needed to evaluate clinically relevant internal and marginal fit in future studies.

Overall, the properties of the 3D-printed resins evaluated for provisional crowns were comparable to those of bis-acryl or self-cure PMMA resins. Among all the materials tested, CT achieved the highest flexural strength, elastic modulus, and Knoop microhardness, along with the lowest surface roughness, water sorption, and solubility. Although the flexural strength and elastic modulus of ND were lower than those of CT, its results were comparable to bis-acryl resin and superior to self-cure PMMA. Additionally, ND exhibited lower surface roughness than both controls, with comparable water solubility but higher water sorption. In terms of mechanical properties, PB showed similar flexural strength, elastic modulus, and Knoop microhardness to bis-acryl and self-cure PMMA resins. However, PB had higher surface roughness, water sorption, and solubility compared with both controls. All resins presented low radiopacity, and cell viability decreased after a 14-day period.

Importantly, the present results are limited to the tested materials and manufacturers continue to advance in developing new photosensitive resins to address deficiencies in certain properties. In addition to using two commercial controls, we adhered to standardized norms for evaluating most of the properties, enabling comparisons with other studies. Further studies comparing different printers, printing parameters, and additional properties—such as color stability, wear resistance, and printing accuracy—are essential to provide a more comprehensive understanding of these materials. This study also demonstrated the feasibility of achieving similar performance in 3D-printed resins using an affordable LCD printer. Based on the properties evaluated, our results suggest that some photosensitive resins for additive manufacturing may offer clinical performance comparable to bis-acryl or self-cure PMMA resins in terms of physicomechanical and biological parameters for fabricating provisional crowns.

## CONCLUSIONS

Of the tested materials, Cosmos Temp (3D resin) had the most favorable overall physicomechanical performance. All materials, including controls, showed low radiopacity, and cell viability decreased in almost all groups after 14 days, with the exception of BR. Within the limitations of this *in vitro* study, the findings suggest that selected photosensitive resins designed for DLP systems can be processed in an affordable LCD printer with physicomechanical performance comparable to bis-acryl and self-cure PMMA resins. However, the low radiopacity of all 3D-printed materials and the time-dependent decrease in cell viability underscore the need for further investigation before any clinical recommendations can be made, particularly regarding radiographic monitoring and long-term biocompatibility.
